# Statement of Retraction: Everolimus ameliorates Helicobacter pylori infection-induced inflammation in gastric epithelial cells

**DOI:** 10.1080/21655979.2026.2728867

**Published:** 2026-10-01

We, the Editors and Publisher of the journal *Bioengineered*, have retracted the following article from publication.

Liu, J., Zhang, F., Zhang, Z., & Zheng, C. (2022). Everolimus ameliorates Helicobacter pylori infection-induced inflammation in gastric epithelial cells. *Bioengineered*, 13(5), 11361–11372. https://doi.org/10.1080/21655979.2021.2018533

Since publication, significant concerns have been raised about the integrity of the data and reported results in the article.

When approached for an explanation, the authors did not respond to our queries and so these serious concerns remain unaddressed.

As verifying the validity of published work is core to the integrity of the scholarly record, we are therefore retracting the article. The authors have been informed of this decision.

We have been informed in our decision-making by our policy on publishing ethics and integrity and COPE guidelines. 

The retracted article will remain online to maintain the scholarly record and will be digitally watermarked on each page as ‘Retracted’. 

